# Corrigendum to “The Effects of Cytokines in Adipose Stem Cell-Conditioned Medium on the Migration and Proliferation of Skin Fibroblasts *In Vitro*”

**DOI:** 10.1155/2016/7692306

**Published:** 2016-11-29

**Authors:** Jiajia Zhao, Li Hu, Jiarong Liu, Niya Gong, Lili Chen

**Affiliations:** Department of Stomatology, Union Hospital, Tongji Medical College, Huazhong University of Science and Technology, Wuhan 430022, China

In the article titled “The Effects of Cytokines in Adipose Stem Cell-Conditioned Medium on the Migration and Proliferation of Skin Fibroblasts* In Vitro*” [1], the pictures used in Figure 1(d) are the same as the pictures used in Figures 2(a1)–2(a4) of another article by the same authors, Li et al., “Side-by-Side Comparison of the Biological Characteristics of Human Umbilical Cord and Adipose Tissue-Derived Mesenchymal Stem Cells,” BioMed Research International, vol. 2013, Article ID 438243, 12 pages, 2013. doi: 10.1155/2013/438243. The same protein microarray analysis of ASC-CM was used in both articles as Figures 2(a) and 6(a), respectively, but different cytokines were marked in the same protein microarray picture. The authors apologize for these errors.

The figures are corrected as follows.

## Figures and Tables

**Figure 1:**
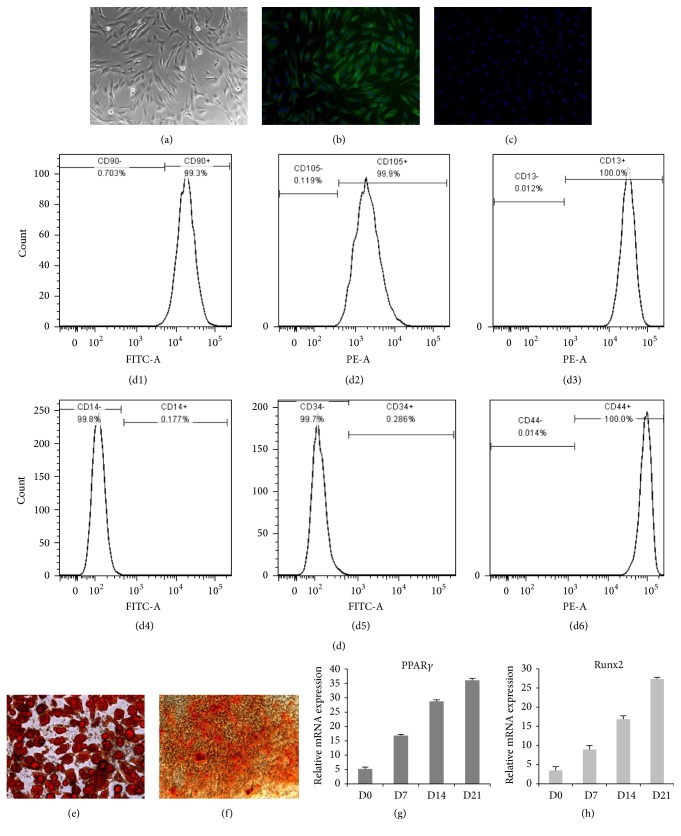
Isolation and identification of human fibroblasts and ASCs* in vitro*. Fibroblasts were spindle-shaped (a) and positive for immunofluorescence staining of vimentin (b) and negative for cytokeratin 15 (c). ASCs were identified by flow cytometry of mesenchymal stem cells markers (d1–d6) and multiple differentiations. Adipogenesis of ASCs was confirmed by Oil Red O staining (e) and gene expressions of PPAR*γ* (g). Osteogenesis was confirmed by Alizarin red staining (f) and gene expression of Runx2 (h).

**Figure 2:**
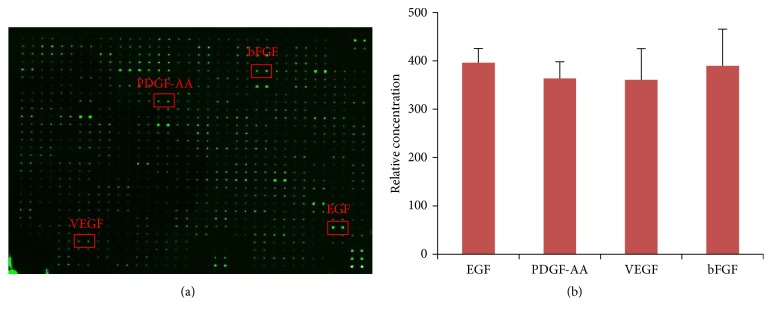
Protein microarray analysis of ASC-CM (a) and relative concentrations of EGF, PDGF-AA, VEGF, and bFGF in ASC-CM (b) (the microarray of ASC-CM was used in our previous published paper [14], but different proteins were highlighted in this paper and previous published paper).
